# Supplementary material for: Reduced Carbon Dioxide by Overexpressing EPSPS Transgene in Arabidopsis and Rice: Implications in Carbon Neutrality through Genetically Engineered Plants
Source: Biology (Basel). 2023 Dec 31;13(1):25. doi: 10.3390/biology13010025 (PMC10813641; doi:10.3390/biology13010025)
Supplement: Supplementary file 1 [file biology-13-00025-s001.zip › Table S1.pdf]

**Table S1.** Primers of the genes from the transcriptome analysis

| Gene Name | Gene ID | Primer Sequence (5' to 3') | Reference |
|---|---|---|---|
| AtUBQ5 | AT3G62250 | F: ATGTGAAGGCGAAGATCCAAGAC | Fang *et al*., 2018 |
| | | R: AGACGGAGGACGAGATGAAGC | |
| AtEPSPS | AT2G45300 | F: AACGCAAGTTATGTCC | Fang *et al*., 2018 |
| | | R: GCAGTTAGTGCCAAG | |
| AtRCA | AT2G39730 | F: CTTCTTGTGTTGTGACGCATACTC | |
| | | R: TCCGTTGCTCTTCTTGTTGCTCTG | |
| AtGAPA | AT1G12900 | F: GTAACCCATCTAATCTCCCCTG | |
| | | R: GGAGCGAGACAGTTAGTAGTAC | |
| AtFBA1 | AT2G21330 | F: GAAGGTATTCTCCTGAAGCCA | Dániel Árpád Carrera *et al*., 2021 |
| | | R: CACCAGACAAGAACATGATTCC | |
| AtSBPASE | AT3G55800 | F: GAAATACACACTGCGATACACC | |
| | | R: GAGTTCTATCGTCGAGGTTGAT | |
| OsACTIN | OS03T0718100 | F: TCCATCTTGGCATCTCTCAG | Jiang *et al*., 2020 |
| | | R: GTACCCGCATCAGGCATCTG | |
| OsEPSPS | OS06T0133900 | F: GCAGTTGGACCATCAGCGAAG | Jiang *et al*., 2020 |
| | | R: CTGTTGAGAAGGATGCGAAAGA | |
| OsRCA | OS11T0707000 | F: GGCTTGACGCTTGCTATTAATT | |
| | | R: AACAAAAACTTGTCATGCCCAG | |
| OsGAPA | OS04T0459500 | F: GTACGACAACGAGTGGGGAT | |
| | | R: GGAAGAAGGAAGAAGTCTGGGG | |
| OsSBPASE | OS04T0234600 | F: GTTTCTTGATAGAGAAAGCCGG | |
| | | R: GATCTCGTTCTTGGAACCGTAG | |
| OsFBA6 | OS11T0171300 | F: ACCGGAAGCAATGGCGTCTGCTA | Fu *et al*., 2022 |
| | | R: TGGTGATGCAATGGTTTTCGCGG | |
